# Point-of-care ultrasound for umbilical venous catheter placement and surveillance in neonates: practical guidance and governance

**DOI:** 10.1007/s00431-026-07392-6

**Published:** 2026-09-09

**Authors:** Bikash Bhojnagarwala, Mahmoud Montasser, Jay Banerjee, Anay Kulkarni

**Affiliations:** 1 Oliver Fisher Neonatal Unit, Medway NHS Foundation Trust, Gillingham, UK; 2 Cantebury Christchurch University, Kent and Medway Medical School, Cantebury, United Kingdom; 3 University Hospital Wishaw, Wishaw, UK; 4 Imperial College Healthcare NHS Trust, London, UK; 5 Imperial College London, London, UK; 6 St George’s Hospital NHS Foundation Trust, London, UK; 7 Royal Brompton and Harefield NHS Foundation Trust, London, UK

**Keywords:** Point-of-care ultrasound, POCUS, Umbilical venous catheter, UVC, Neonates, Placement, Surveillance, Guidance, Governance

## Abstract

Umbilical venous catheters (UVCs) are essential in neonatal intensive care, yet malposition and early migration are common and can cause serious hepatic or cardiac injury. We set out a structured, image-based approach to UVC placement and surveillance using point-of-care ultrasound (POCUS). We undertook a targeted narrative review of literature from 2005 to 2025, drawing on comparative studies, systematic reviews, meta-analyses, and society guidance on neonatal POCUS and vascular access. Findings were synthesised into a continuous bedside workflow covering placement, real-time correction of malposition, surveillance, and governance. POCUS directly visualises the UVC tip at the inferior vena cava–right atrial junction and detects portal, hepatic, and intracardiac malposition more reliably than radiography (reported sensitivity and specificity for cavoatrial localisation > 90% vs 60–70%). Tip migration occurs in 50–90% of neonates within the first 72 h, so a single confirmatory scan at insertion is insufficient. A three-view protocol (situs, subcostal IVC–RA, coronal/oblique) combined with a multi-probe approach reduces misclassification: the curvilinear probe maps the intrahepatic course, the linear probe confirms the tip, and the phased-array probe is reserved for cardiac views. Operator-dependent ultrasound artefact misidentification is a key clinical pitfall that requires multi-planar confirmation and explicit competency standards.

* Conclusion*: A three-view, multi-probe POCUS protocol improves UVC tip confirmation, allows earlier correction of malposition, and detects migration sooner than repeat radiography. Safe local adoption requires recognition of operator-dependent artefact pitfalls within a structured training and governance framework.

**Supplementary Information:**

The online version contains supplementary material available at https://doi.org/10.1007/s00431-026-07392-6.

**Table:** 

**What is Known:** • *Radiography for UVC tip confirmation is indirect and frequently misclassifies portal sinus malposition*. • *Tip migration occurs in up to 90% of neonates within 72 h, exposing infants to ongoing hepatic and cardiac risk*.		
**What is New:** • *A structured three-view, multi-probe POCUS protocol that directly localises the UVC tip and distinguishes portal, hepatic, and intracardiac malpositions at the bedside*. • *Recognition and mitigation of operator-dependent pitfalls, addressed alongside placement, surveillance, and governance as one practical bedside workflow*.		

## Introduction and background: why UVC positioning matters

Umbilical venous catheters (UVCs) give rapid central venous access to critically ill term or preterm infants and are a mainstay of neonatal intensive care. They are usually inserted soon after birth, often in haemodynamically unstable babies needing urgent vascular access for parenteral nutrition and essential medications.

Correct positioning at insertion does not guarantee safety. Serial ultrasound studies have shown tip migration in 50–90% of neonates during the first 24–72 h after insertion [1–3]. Xie et al. demonstrated that 15% of these displacements had clinical implications, and daily POCUS surveillance allowed early detection and avoided 486 radiographs [3]. The catheter can migrate distally into the right atrium or proximally into the portal venous system, and neither is reliably detected on routine radiographs.

Hermansen and Hermansen documented complications associated with malposition, including thromboembolism, arrhythmia, pericardial effusion and tamponade, infection, hepatic injury, and portal venous thrombosis [4]. More recently, Young et al. reinforced the link between UVC malposition and neonatal morbidity and outlined how bedside imaging can clarify tip location and guide repositioning [5].

This review sets out a practical, image-based approach to UVC localisation, real-time correction during insertion, ongoing surveillance, and the governance arrangements needed to support safe local adoption.

## Why ultrasound outperforms radiography for UVC tip localisation

Thoracoabdominal radiography has long been the routine method of confirming UVC tip position, but it is indirect: the tip location is inferred from vertebral and diaphragmatic landmarks rather than directly visualising the inferior vena cava–right atrial (IVC–RA) junction. Several comparative studies have shown that agreement between radiography and ultrasound is poor [6–8]. Franta et al. found that only 38% of UVCs deemed acceptable on radiographs were optimally positioned on ultrasound, with migration in nearly 50% within the first week [6]. Simanovsky et al. reported similar inconsistencies in 75 neonates [7]. A meta-analysis of 14 studies reported pooled sensitivity of 90% and specificity of 82% for radiographic verification and concluded that ultrasound should be considered the reference standard for tip localisation, particularly in premature infants [8]. Because tip position changes within the first 2 to 3 days [1–3], a single radiograph at insertion does not reliably reflect catheter position when parenteral nutrition or inotropes are subsequently administered, leaving radiography alone both imprecise and inadequate for ongoing care.

POCUS shows the catheter tip directly within the IVC and confirms its position at the IVC–RA junction [8–10]. Michel et al. compared the two techniques in 60 neonates and reported markedly higher sensitivity and specificity for ultrasound (93.3% and 95.6% versus 66.7% and 63.0%, *p* < 0.001) [9]. D’Andrea et al. showed that real-time ultrasound guidance reduced malposition from 75.9 to 9.6% (OR 29.5) [11]. A 2025 meta-analysis of 1322 neonates found that ultrasound halved the risk of malposition compared with radiographic guidance (RR 0.51, 95% CI 0.37–0.70 in RCTs) [12]. Beyond malposition, ultrasound reduces radiation exposure: Rossi et al. reported that the proportion of neonates requiring multiple radiographs fell from 38.7 to 9.9% with bedside ultrasound [13]. It can also serve as a rescue technique when blind insertion has failed: Kozyak et al. achieved successful central placement in 72% of neonates with congenital heart disease whose catheters had previously failed blind insertion [14]. The principal differences between the two modalities are summarised in Supplementary Table 1.

## The umbilical venous pathway: where catheters go astray

**Fig. 1:**
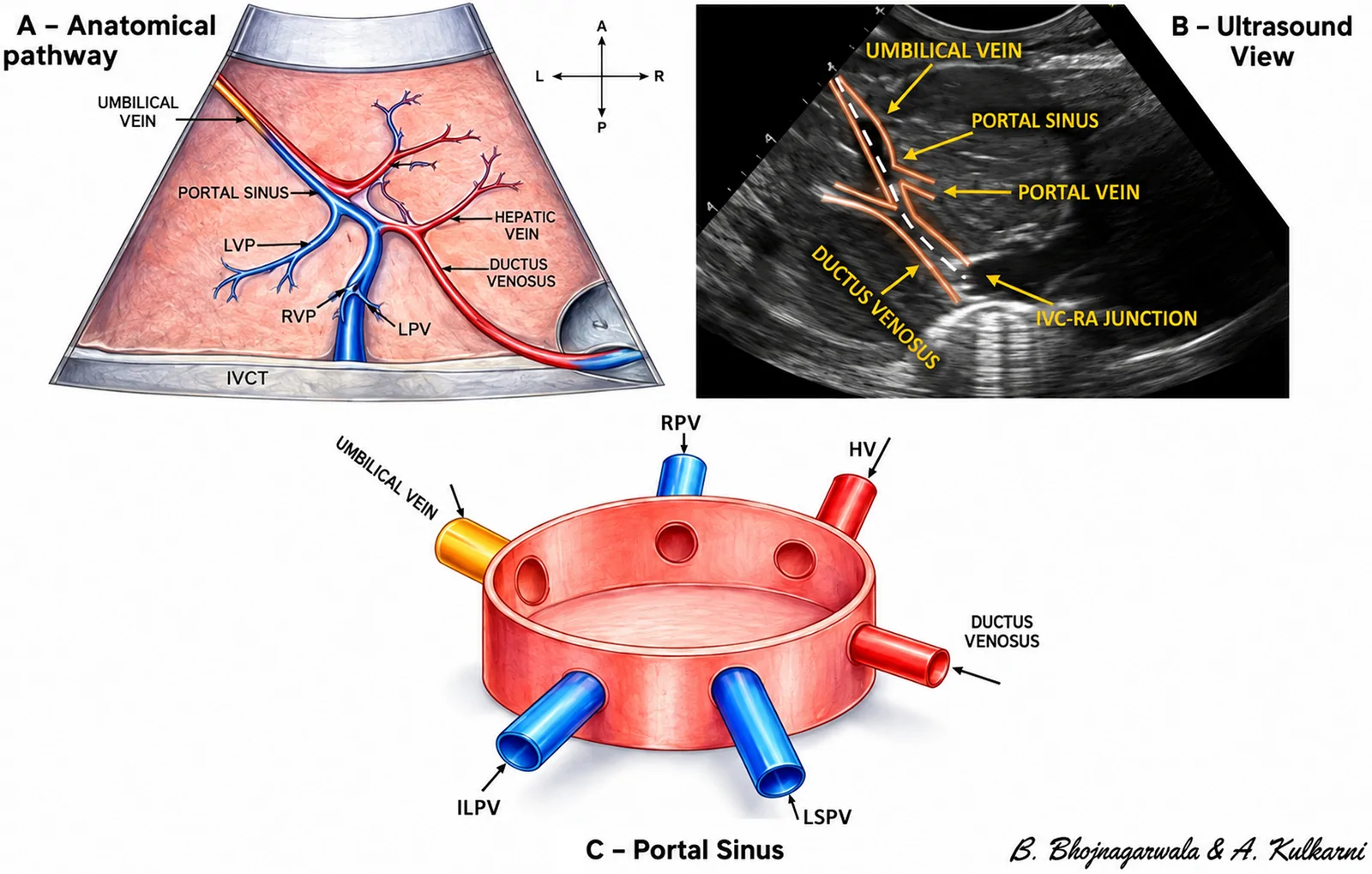
Schematic of the umbilical venous pathway with ultrasound correlation illustrating the portal sinus branching points where the catheters deviate most often

Knowing the venous pathway that the catheter must follow and the sites at which it is likely to deviate is essential to understanding the position of the UVC. A correctly placed UVC will follow the venous pathway from the umbilical vein to the portal sinus, into the ductus venosus, into the inferior vena cava (IVC), and into the IVC–right atrial (RA) junction (Fig. 1).

The most common malposition occurs at the portal sinus, where the umbilical vein joins the portal venous system. From the portal sinus, the ductus venosus continues towards the IVC while the right and left portal veins distribute blood to the liver. The acute angle at this junction often deflects the catheter tip into the portal system rather than the ductus venosus [15–17]. A catheter that fails to enter the ductus venosus and lodges at the portal sinus is anatomically and haemodynamically vulnerable: Small movements (from handling, abdominal pressure, or drying of the umbilical stump) can redirect the tip into a segmental portal vein, causing intrahepatic malposition and progressive hepatic injury [15, 16]. Crucially, the portal sinus is not a great vessel: it lacks the high blood flow of the IVC needed to dilute concentrated solutions, and any infusion delivered here drains directly into the liver. A UVC tip in the portal sinus is therefore not a safe route for parenteral nutrition, hypertonic glucose, vasoactive drugs, or other hyperosmolar infusions, all of which risk hepatocellular injury and portal venous thrombosis when delivered at this site [4, 5].

This position is easily missed on radiography. A tip projected to T10–T11 is conventionally interpreted as a satisfactory ‘low midline’ position, yet ultrasound frequently shows the same tip sitting at the portal sinus rather than within the IVC (Fig. 1C). Ultrasound confirmation of ductus venosus entry is therefore the only reliable check at the bedside.

On ultrasound, the IVC appears as a linear hypoechoic structure behind the liver that drains into the right atrium. The tip should sit at the IVC–RA junction, at or just below the lower border of the right atrium [4]. A tip within the right atrium risks arrhythmia and pericardial tamponade; a tip in the portal system risks hepatic injury and portal venous thrombosis.

**Table:** 

Key practice points
➤ A correctly placed UVC follows the path UV → portal sinus → ductus venosus → IVC → IVC–RA junction
➤ The most common site of deviation is the portal sinus, where the umbilical vein joins the portal venous system [15–17]
➤ Ensuring that the tip of the UVC enters the ductus venosus is an essential step in correctly placing the UVC catheter [15, 16]
➤ The portal sinus is not a great vessel: A tip lodged here is unsafe for parenteral nutrition, hypertonic dextrose, vasoactive drugs, or other hyperosmolar infusions [4, 5]
➤ The target tip position is the IVC–RA junction [4]

## A practical approach to UVC assessment with ultrasound

Four things determine the quality and reliability of a UVC scan: the probe, image optimisation, the views obtained, and the operator’s recognition of artefacts and pitfalls. A clear cotside set-up, with defined roles for the operator and assistant, helps with both safety and efficiency [18].

### Probe selection

**Fig. 2:**
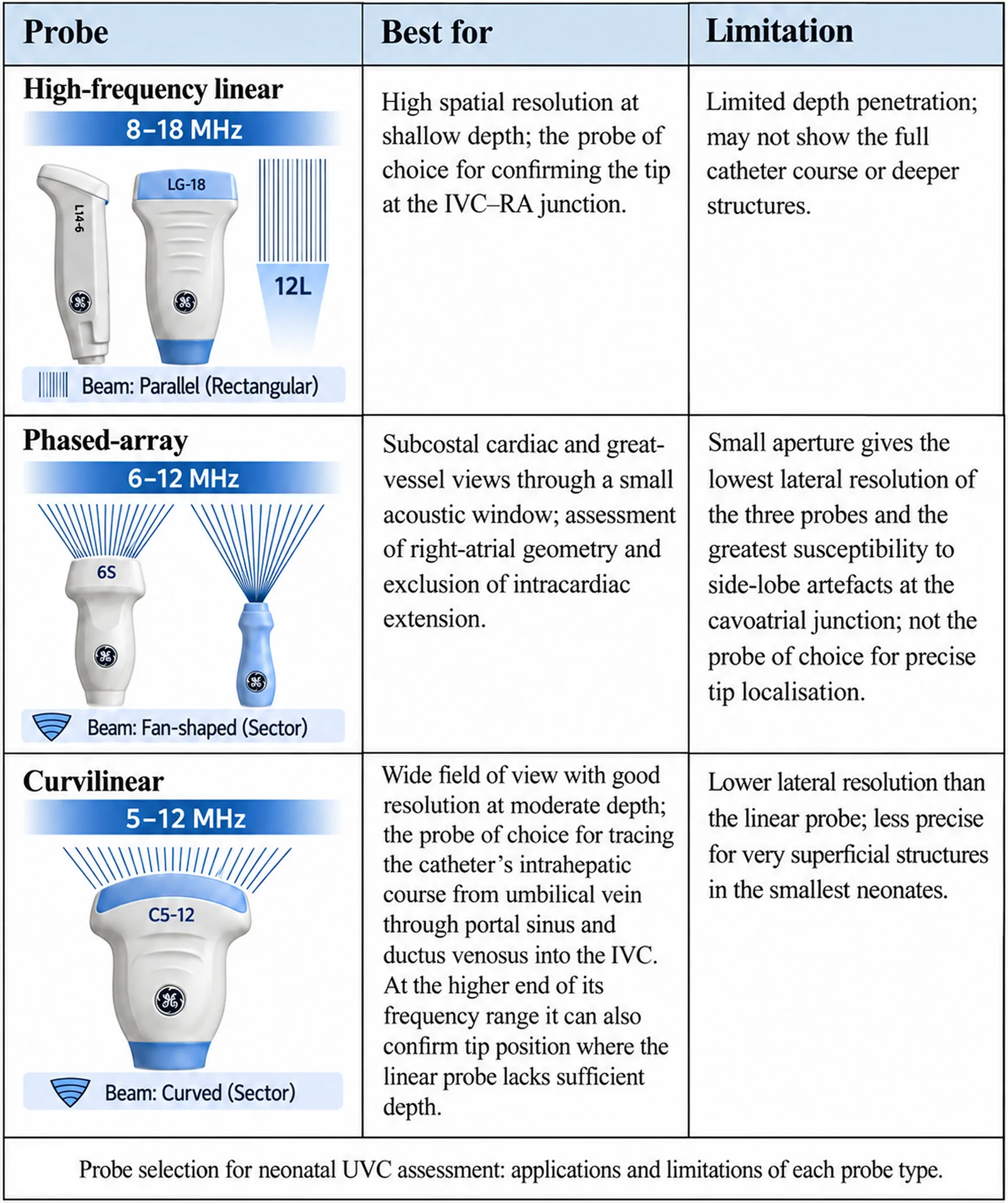
Probe selection for neonatal UVC assessment: applications and limitations of each probe type

Three probe types are commonly used for neonatal UVC assessment; their characteristics, applications, and key limitations are summarised in Fig. 2.

No single probe gives a complete picture of the UVC course and tip, and the limitations of each become more clinically important as the catheter approaches the IVC–RA junction. A staged, multi-probe approach works best in practice (Fig. 2). Use the curvilinear probe first to map the catheter’s intrahepatic course from the umbilical vein, through the portal sinus and ductus venosus, into the IVC. Switch to a high-frequency linear probe to confirm the tip precisely at the IVC–RA junction, where its superior near-field resolution shows the catheter and the cavoatrial anatomy in sharp detail [9, 15–17]. The phased-array probe is reserved for subcostal cardiac views (useful for excluding intracardiac extension or assessing right atrial geometry) rather than for primary tip identification.

### Image optimisation

Set the depth to include the full intrahepatic course and the IVC–RA junction, usually 4 to 6 cm. Going deeper than this loses resolution; setting it too shallow may leave the cavoatrial junction off-screen [9, 15, 16].

Adjust the gain so that the vessels appear black (anechoic) and the echogenic catheter tip stands out clearly. If the gain is too high, anatomical detail is washed out; if it is too low, the catheter itself can be lost [15, 16]. Sector width and focal zone position should be set to maximise contrast at the depth of interest [15, 16].

Avoid pressing too hard with the probe. Compression of the IVC can distort the anatomy or even shift the catheter position [15]. When scanning before the line has been secured, use sterile gel.

### A structured three-view approach

**Fig. 3:**
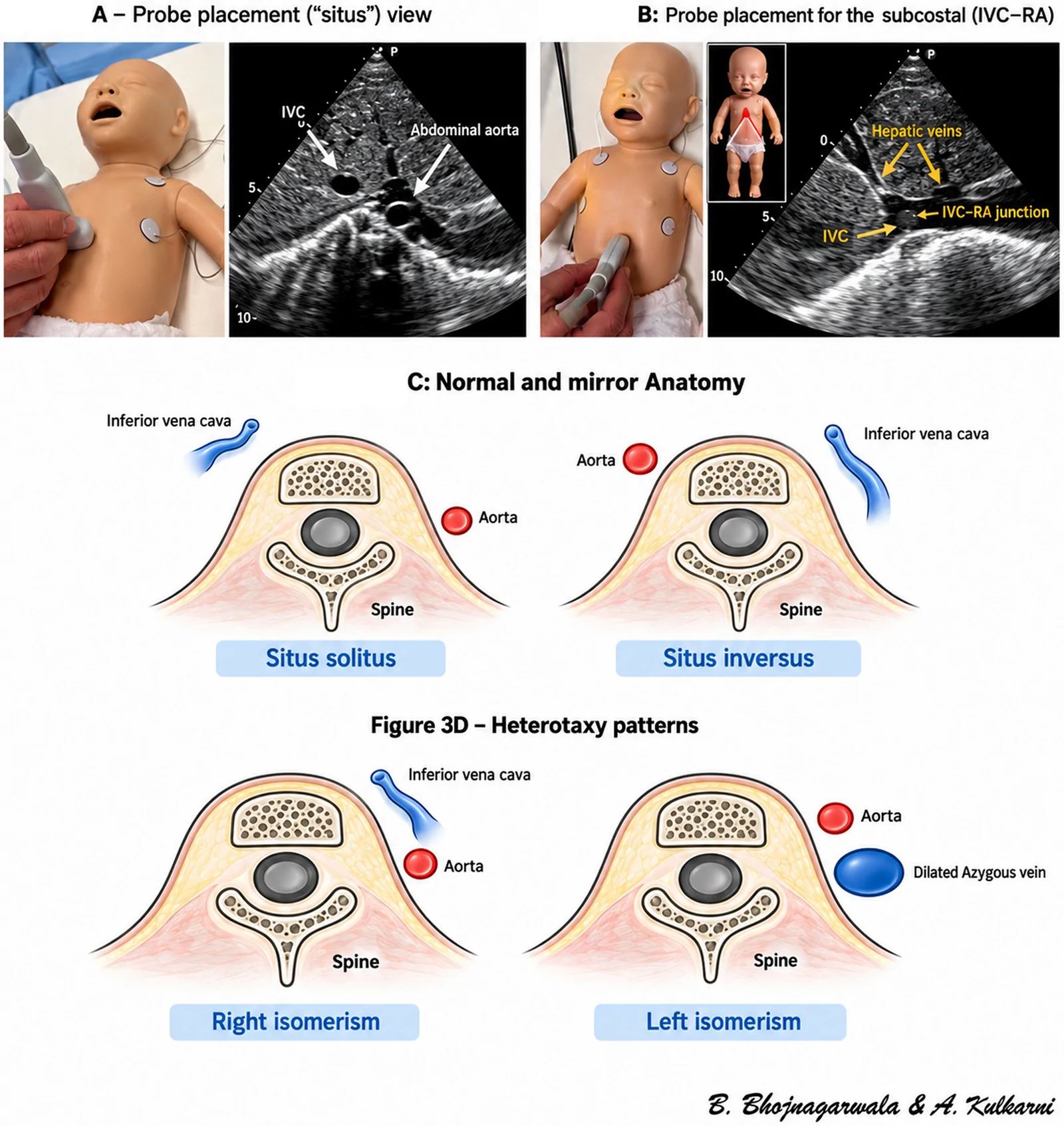
Ultrasound views and anatomical variants relevant to neonatal UVC assessment. **A** Transverse abdominal (‘situs’) view. **B** Subcostal view. **C** Normal and mirror image vascular anatomy (situs solitus and situs inversus). **D** Heterotaxy patterns with altered systemic venous anatomy

A three-view protocol (Fig. 3) reliably localises the tip:

#### Transverse upper abdominal view (situs)

Place the ultrasound probe in a transverse position below the costal margin with the probe marker pointing towards the infant’s left side. Assess the situs of the major vessels in the abdominal area; the aorta should be visible to the left of the spine while the IVC should be to the right (Fig. 3A).

#### Subcostal longitudinal view (IVC–RA)

Place the probe below the xiphisternum in a longitudinal plane with the probe marker pointing towards the infant’s head. The bright echogenic tip of the catheter should be visible at the junction of the IVC and RA (Fig. 3B).

#### Coronal/oblique liver view

Use this view when the first two are inconclusive—it confirms the catheter’s course through the ductus venosus and helps exclude portal venous malposition. Visualising the ductus venosus entering the IVC is the most reliable single sign of correct placement [9, 15, 17].

### Techniques, pitfalls, and artefacts

POCUS for UVC localisation requires more than greyscale imaging in two planes. Active confirmation techniques and explicit recognition of artefacts that can mislead are both essential. The ‘*see it, move it, flush it*’ framework summarises the bedside workflow: *See* the catheter directly on greyscale, *move* the line gently to confirm what shifts with the catheter, and then deliver a saline *flush* to confirm intravascular position.

#### Probe angulation

**Fig. 4:**
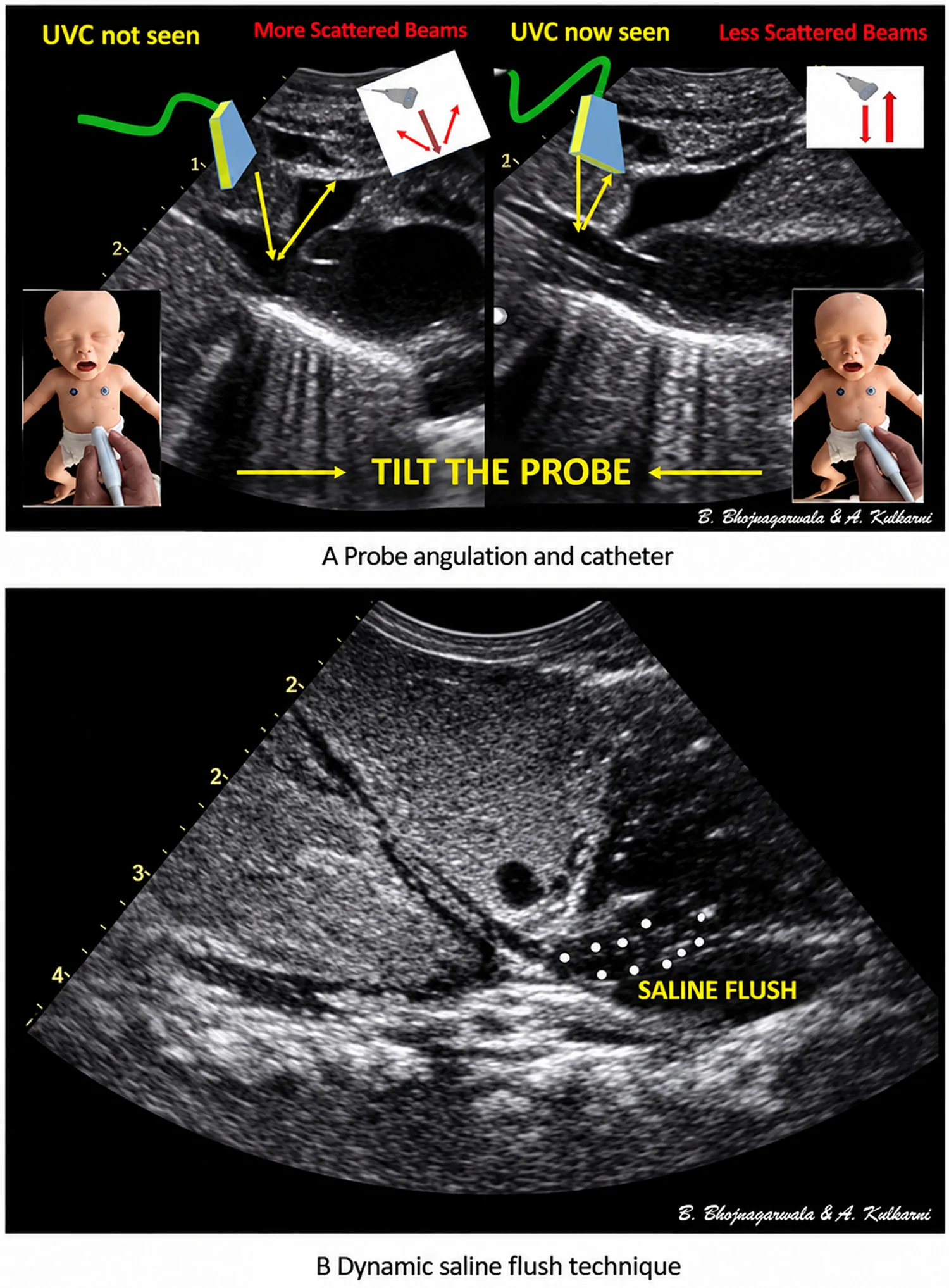
Techniques to optimise UVC visualisation and tip confirmation. **A** Probe angulation to improve catheter tip visibility. **B** Saline flush to confirm intravascular catheter position

Small adjustments in probe angle, usually only a few degrees, bring the catheter and the IVC into the same imaging plane, eliminating apparent ‘tip drop-off’ caused by the catheter slipping out of the slice (Fig. 4A). When the tip cannot be visualised in a single plane, slow rotation and tilt usually recover it before any further intervention is considered.

#### Saline flush confirmation

A 1–2 mL bolus of saline injected via the catheter produces a clear stream of echogenic microbubbles within the vessel at the tip (Fig. 4B). Bubbles appearing at the IVC–RA junction confirm both vascular position and tip location. Bubbles appearing within the liver parenchyma indicate hepatic infiltration or extravasation [19]; bubbles within the right atrium confirm intracardiac tip position. Saline flush is particularly valuable when greyscale alone leaves uncertainty about whether a bright echo represents the catheter or an artefact.

#### Side-lobe artefacts

Phased-array probes are particularly prone to side-lobe artefacts: Their tightly grouped crystals make off-axis beam components more significant, and a side-lobe encountering a strong reflector produces a ‘ghost’ image projected onto the central beam path that can mimic a correctly positioned catheter tip. The risk is greatest near the cavoatrial junction, where the diaphragm, calcified ligamentum venosum, and atrial wall act as strong reflectors. Tissue harmonic imaging suppresses side-lobe artefacts and should be activated when in doubt [20].

#### Acoustic shadowing and mirror artefact

Acoustic shadowing distal to bowel gas or rib can obscure the true catheter tip, particularly in the subcostal view. Mirror artefact at the diaphragm–lung interface can produce a duplicate echo above the diaphragm that mimics intracardiac extension. Both pitfalls are mitigated by changing probe position or angulation rather than relying on a single view [20].

#### Distinguishing a ghost from a true tip

A bright linear echo close to the IVC–RA junction may represent the catheter tip, a ghost from an off-axis reflector, or a fold in the atrial wall. Three checks reliably distinguish these: (i) confirmation in at least two orthogonal planes; (ii) gentle catheter movement: the true tip moves with the line, a ghost does not; (iii) saline flush: microbubbles appear only at the true intravascular tip. No single view should be accepted as definitive; multi-planar confirmation is essential [15, 17, 20]. Failure to recognise these pitfalls may delay correction of a true malposition that remains undetected behind a misidentified ghost [19].

**Table:** 

Key practice points
➤ The three-view approach to UVC tip localisation improves the accuracy of anatomical localisation and reduces misclassification of the tip position of UVCs [15–17]
➤ The transverse abdominal view localises the vessels to determine the situs of the catheter—the aorta should be on the left and IVC on the right of the spine [15, 16]
➤ The subcostal longitudinal view is the reference view for confirming the tip at the junction of the IVC and RA [7, 9]
➤ Multi-planar assessment allows for the correct classification of the tip of UVCs relative to both portal (liver) and hepatic vessels [15–17]
➤ Various techniques, such as angulating the probe and using a saline flush, are useful in addition to greyscale imaging of the tip of the UVC catheter **‘See it, move it, flush it’:** *See the catheter directly, optimise probe alignment, and then confirm with a saline flush*

## Interpreting UVC position on ultrasound: what you see and what to do

**Fig. 5:**
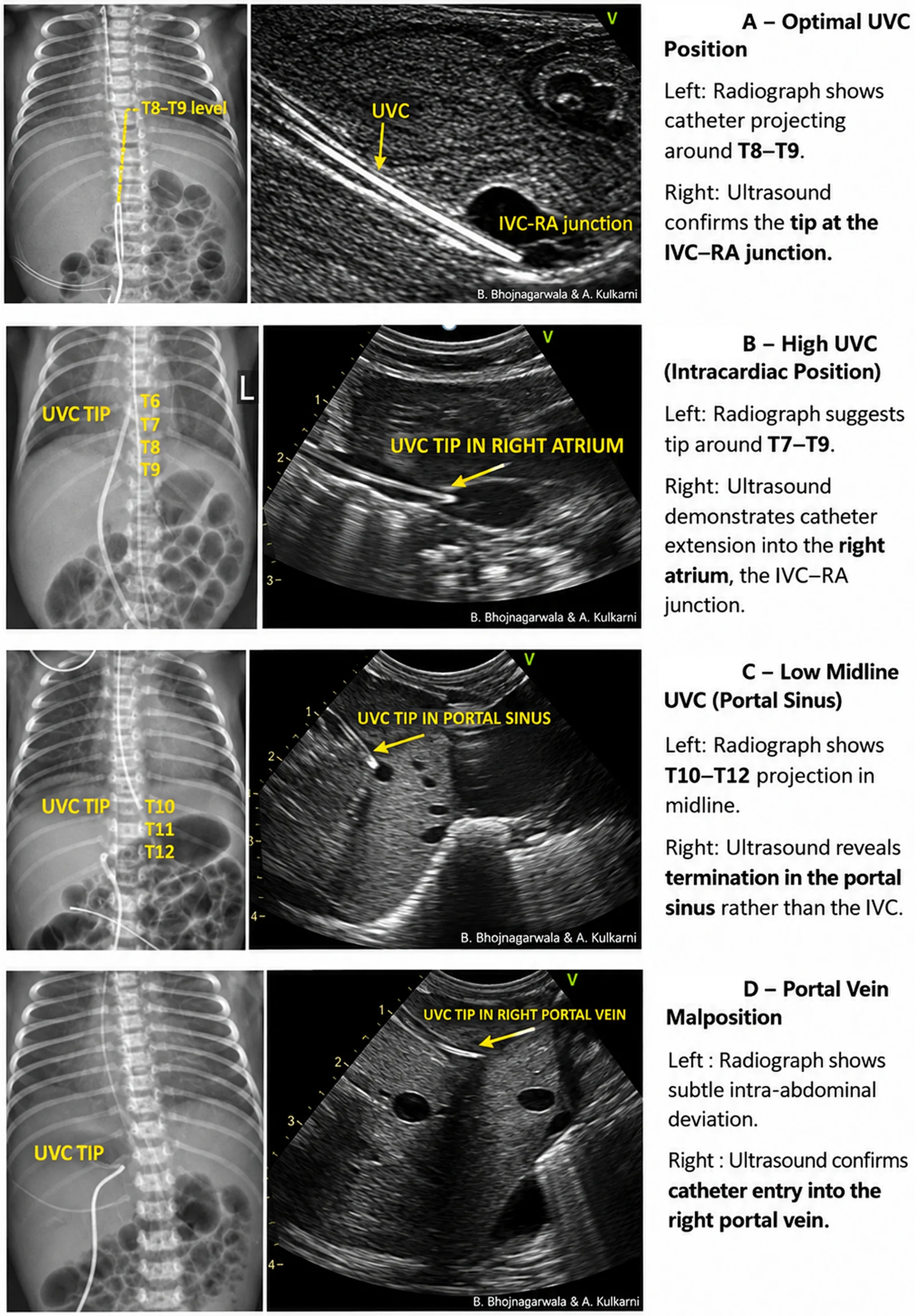
Radiographic versus ultrasound assessment of tip of UVC catheter

Because ultrasound shows the tip directly, the position can be classified and acted on immediately, without waiting for a radiograph. Table 1 summarises the four most common tip positions and their management, with corresponding ultrasound appearances illustrated in Fig. 5. Of these positions, the ‘low midline/portal sinus’ position warrants particular care for the reasons set out earlier.

**Table 1:** Common UVC tip positions identified on ultrasound (as illustrated in Fig. 5) with clinical implications and recommended management

Position	Ultrasound appearance	Clinical risk	Action
Optimal (IVC–RA junction) (Fig. 5A)	Echogenic line ending at the cavoatrial junction, just below the RA border	Lowest complication risk [4]	Use as intended; re-scan within 24 h
Low midline/portal sinus (Fig. 5B)	Tip below diaphragm, not entering the IVC; appears midline on radiograph but in the portal sinus on ultrasound	Portal venous thrombosis; hepatic injury; not safe for use	Reposition under POCUS; avoid hyperosmolar infusions
High/intracardiac (RA) (Fig. 5C)	Tip within the right atrium; may move with the cardiac cycle	Arrhythmia; pericardial effusion; tamponade [4]	Withdraw into the IVC under ultrasound guidance
Portal or hepatic branch (Fig. 5D)	Catheter deviating into a portal vein or hepatic parenchyma	Hepatic necrosis; thrombosis; extravasation [4, 19]	Do not use; remove and re-site

## POCUS-guided UVC insertion and manipulation

Real-time POCUS lets the operator watch the catheter advance and confirm correct positioning as it happens. Depth formulae are useful, but seeing the catheter pass through the ductus venosus and into the IVC remains the most reliable confirmation of placement.

Several studies support ultrasound-guided insertion. Kishigami et al. used gentle upper-abdominal compression to align the umbilical vein with the ductus venosus, achieving correct positioning in 93% of 15 neonates without complications [15]. Tomaszkiewicz et al., in a series of 305 neonates, reported insertion success rising from 83.8 to 89.1% with real-time guidance [16]. Rubortone et al. showed that NICU staff training increased ultrasound use from 15.3 to 89.2% of insertions and more than doubled the rate of IVC–RA junction placement [17]. In a randomised controlled trial, Mishra et al. found that ultrasound guidance did not reduce insertion failure but improved tip localisation sensitivity and shortened time to confirmation compared with chest radiography [21]. These findings are consistent with a 2024 systematic review concluding that ultrasound guidance for neonatal vascular access improves first-attempt success and reduces mechanical complications compared with landmark-based techniques [22].

### Ultrasound-guided correction of portal deviation

If the catheter keeps entering the portal system, this reflects misalignment at the ductus venosus junction. Use a curvilinear or phased-array probe to visualise the portal sinus, ductus venosus, and IVC together so that the position can be corrected under continuous ultrasound view.

Gentle external pressure on the liver near the portal sinus can help redirect the catheter towards the ductus venosus by altering the angle of the intrahepatic veins. The catheter tip can then be partially withdrawn and re-inserted slowly until successful entry into the IVC is achieved (Supplementary Fig. 1A, B).

Avoid excessive force during advancement. Resistance often reflects unfavourable anatomy or partial closure of the ductus venosus, and in either case, the catheter should be withdrawn and re-sited rather than forced.

### Avoidance of ‘railroading’ a second catheter

There is no evidence to support inserting a second catheter alongside an existing UVC (‘railroading’), and the practice increases the risk of infection and vascular injury. If the first catheter cannot be advanced to the IVC, it should be withdrawn and re-sited rather than supplemented with a second line.

**Table:** 

Key practice points
➤ Real-time ultrasound guidance has been shown to assist with the correct placement of the catheter relative to the central venous system. Studies reporting on the outcomes of UVC placement with ultrasound guidance have demonstrated that the rate of correctly placed catheters is higher with ultrasound guidance relative to those placed blindly [15–17, 21]
➤ The most common site of deviation of the catheter from the portal sinus in relation to the central venous system is the junction between the portal sinus and the ductus venosus. Therefore, image guidance to the catheter to that region can help to direct the catheter into the proper placement relative to the ductus venosus [15, 16]
➤ Applying gentle external pressure to the liver near the portal sinus has been shown to redirect the catheter into the ductus venosus [15]
➤ Should it not be possible to achieve central venous alignment with the UVC catheter, the catheter should be removed and re-sited rather than repeatedly pushed against the venous system or ‘railroaded’ into the central venous system with a second line

## Surveillance, migration, and early recognition of complications

A correctly positioned UVC at insertion does not stay safe for long: tip migration occurs in 50–90% of neonates, most of it within the first 48 to 72 h [1, 6].

Because POCUS uses no ionising radiation, it can be repeated as often as clinically required. In Xie’s cohort, daily POCUS detected displacement in 94% of neonates, and 15% of these displacements were clinically relevant [3]. Sobczak et al. reported displacement in 68% of catheters during their surveillance period, with serial scans frequently prompting management changes [2].

Practical surveillance should include a routine rescan within the first 24 h after insertion and then further scans during the high-risk first few days and whenever clinically indicated. Common triggers for an additional scan include unexplained deterioration, new difficulty with infusion, or any change in catheter function.

**Table:** 

When to rescan the UVC
Routinely within 24 h of insertion, irrespective of initial optimal placement
Every 48 h while the UVC remains in situ (pragmatic safety practice)
After any catheter manipulation or documented change in external catheter length
In the event of unexpected clinical deterioration, hypotension, or arrhythmia
In the event of new abdominal distension or isolated ascites; assume catheter-related extravasation until proven otherwise^12^
Loss of previously aspirated blood return

## Governance, training, competency, and documentation in neonatal UVC POCUS

Safe integration of POCUS into UVC care depends on defined competency, structured documentation, and local quality assurance. The ESPNIC and AAP guidance recommend that POCUS be delivered within institutional arrangements that set scope of practice, define competency, and include image review [23, 24]. The Canadian Paediatric Society statement adds particular emphasis on educational pathways and credentialing [25].

Competency involves more than acquiring an image; it requires correct interpretation and safe action. Kochan et al. found that simulation-enhanced training improved both image acquisition and interpretation among NICU staff, although procedural competency on real patients took more than ten supervised scans to develop [18].

Governance arrangements should cover secure image storage, structured documentation of each scan, and a route for peer review of saved images. Building POCUS into existing UVC guidance, rather than running it as a parallel workflow, makes it more sustainable and reduces variation in how it is used.

Practical implementation tools, including a bedside checklist, a documentation template, and a suggested competency framework, are provided in the supplementary appendices.

## Limitations

This review has limitations. We did not use a systematic search; studies were selected on expert judgement. Most included studies are single-centre and observational, with few randomised controlled trials. Heterogeneity in populations, POCUS protocols, and definitions of optimal tip position limits direct comparison, and variation in operator experience further limits generalisability across centres. The accuracy of POCUS for UVC tip localisation is itself operator-dependent: Artefact-related misidentification, particularly side-lobe ghosting near the cavoatrial junction, can falsely reassure inadequately trained operators and remains a real risk, mitigated only by structured training, multi-plane confirmation, and awareness of standard ultrasound physics principles [18, 20]. Despite these limitations, our recommendations align with the current evidence base and with international neonatal and paediatric society guidance.

## Final summary: ultrasound as the contemporary standard for UVC placement and surveillance

UVC safety rests on two things: accurate placement at insertion and early detection of migration, which is common in the first 24 to 72 h. POCUS shows the catheter trajectory and the tip at the IVC–RA junction directly and confirms placement with greater accuracy than radiography [12]. The IVC–RA junction remains the safest anatomical target for sustained central infusion [7, 9].

POCUS is also useful beyond initial confirmation: it allows real-time correction of portal deviation during insertion and detects complications, such as catheter-related extravasation, that may otherwise be missed [19]. Structured surveillance during the high-risk early period, together with appropriate training and governance, is essential for safe implementation [23–25]. These elements shift neonatal vascular access from landmark-based estimation towards image-guided practice.

POCUS for UVC care is an operator-dependent skill. Accuracy depends as much on anatomical familiarity and artefact recognition as on having the equipment to hand. Side-lobe artefacts and ghost images can mimic a correctly positioned tip or hide a malpositioned one, and reliance on a single plane or a single probe substantially increases this risk. Confirmation in two planes, a multi-probe approach, and simple dynamic manoeuvres (‘see it, move it, flush it’) together reduce the chance of misidentification. Safe local adoption therefore needs more than equipment: it needs a training pathway, supervised competency sign-off, and governance to embed these standards across the unit.

**Table:** 

Summary practice points
UVC tip position is dynamic; migration occurs in 50–90% of neonates within the first 72 h [1–3]
A radiographic ‘low midline’ appearance does not exclude portal sinus placement; ultrasound is required to confirm IVC entry [6–8]
The IVC–RA junction is the only consistently safe anatomical target for sustained central infusion [7, 9]
A three-view POCUS protocol (situs, subcostal IVC–RA, coronal/oblique) provides anatomical clarity and reduces misclassification [15, 16]
Gentle hepatic compression under continuous ultrasound guidance can facilitate redirection through the ductus venosus when portal deviation occurs [15, 16]
Reassess within 24 h of insertion and after any clinical change or catheter manipulation [1, 6]
Implementation of this technique requires training, credentialing, image archiving, and audit within a governing framework [23, 24]

## Supplementary Information

Below is the link to the electronic supplementary material.

ESM 1 (PDF 4.14 MB)

## Data Availability

No datasets were generated or analysed during the current study.

## Declarations

### AI assistance declaration

AI-assisted tools (Claude, Anthropic) were used for paraphrasing and language editing during manuscript preparation. No scientific content, data, figures, or conclusions were generated using AI. All references were manually verified against primary sources. The authors take full responsibility for the accuracy and integrity of all content.
