# Supplementary material for: Point-of-care ultrasound for umbilical venous catheter placement and surveillance in neonates: practical guidance and governance
Source: Eur J Pediatr. 2026 Sep 9;185(10):734. doi: 10.1007/s00431-026-07392-6 (PMC13558298; doi:10.1007/s00431-026-07392-6)
Supplement: Supplementary file 1 [file 431_2026_7392_MOESM1_ESM.pdf]

Supplementary Material

Point-of-Care Ultrasound for Umbilical Venous Catheter Placement and Surveillance in Neonates: Practical Guidance and Governance

Bhojnagarwala, Montasser, Banerjee, Kulkarni — European Journal of Pediatrics

Contents

Supplementary Figure 1   Real-time ultrasound-guided correction of UVC deviation into the portal venous system.

Supplementary Figure 2   Practical Guidance Flowchart (A3 landscape — final pages).

Supplementary Table 1   Comparison of radiography and POCUS for UVC tip localisation and surveillance.

Supplementary Appendix 1   Bedside POCUS UVC Checklist.

Supplementary Appendix 2   Documentation Template.

Supplementary Appendix 3   Minimum Competency Framework.

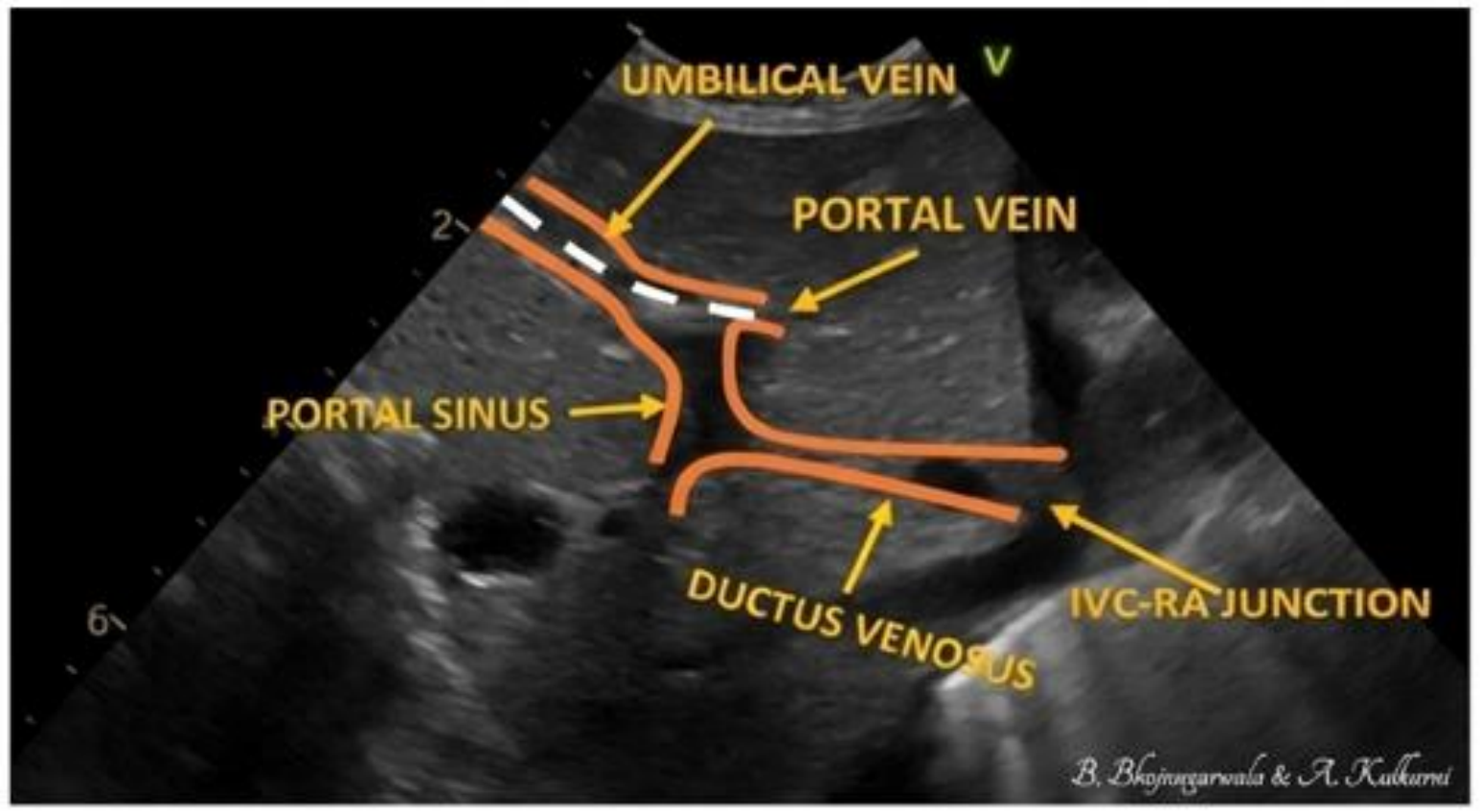

Figure 1A.

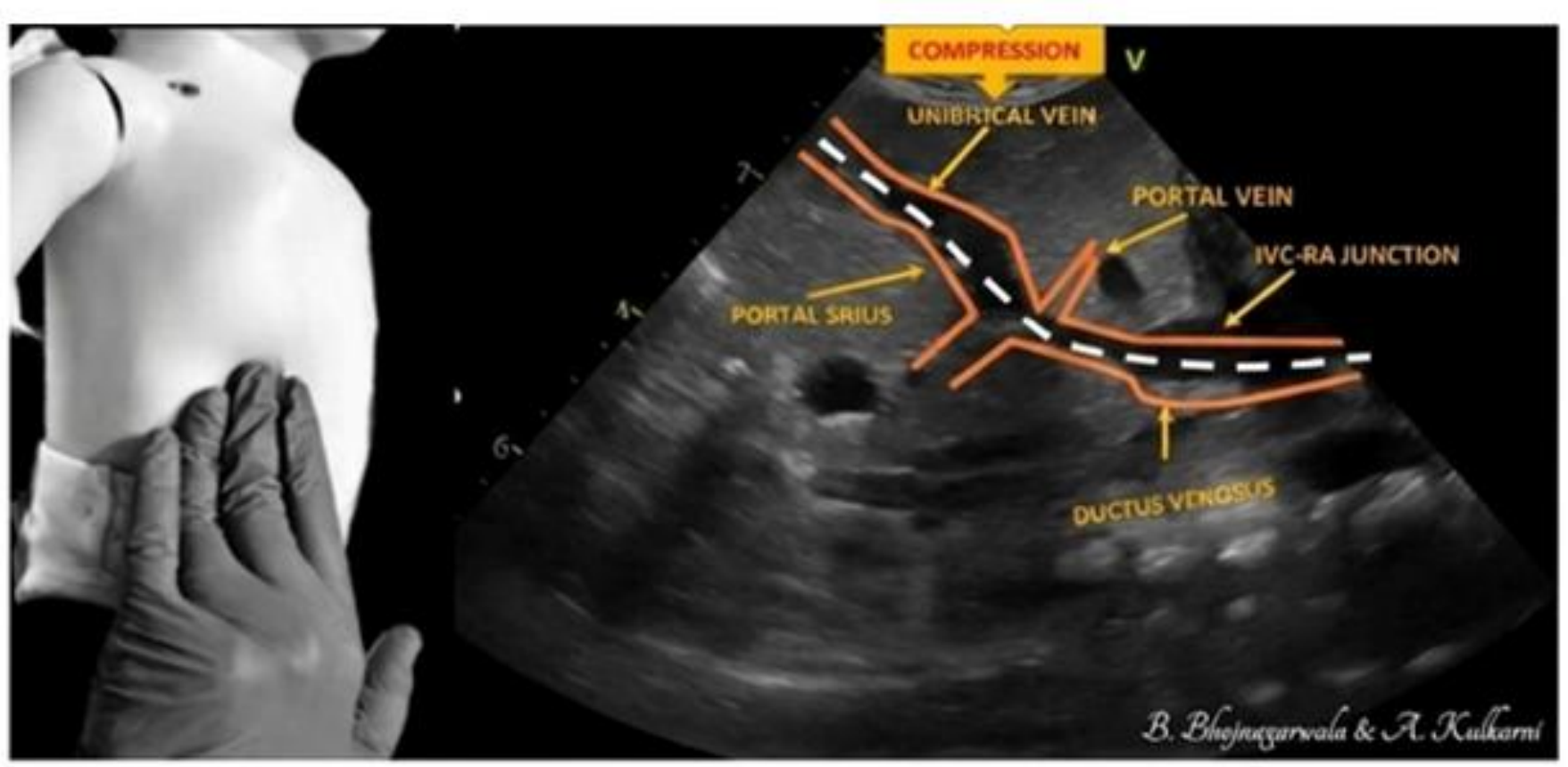

Figure 1B.

Supplementary Figure 1. Real-time ultrasound-guided correction of umbilical venous catheter deviation into the portal venous system. **(A)** Initial ultrasound image showing the catheter tip deflected into a portal vein rather than entering the ductus venosus — a position carrying significant risk of hepatic injury and unsafe for parenteral nutrition, hypertonic glucose, or vasoactive infusions. **(B)** Repeat image of the same neonate after partial withdrawal and re-advancement under real-time ultrasound guidance, demonstrating the catheter now passing through the ductus venosus into the inferior vena cava, with the tip resting at the IVC–right atrial junction.

Supplementary Figure 2 – Practical Guidance Flowchart : Bhojnagarwala et al. (2026) · European Journal of Pediatrics

POCUS for Umbilical Venous Catheter (UVC) Placement & Surveillance in Neonates

■ Optimal / Proceed	■ Caution / Reposition	■ Withdrawal Required	■ Do Not Use / Remove	■ Protocol / Process	■ Governance

PHASE 1 — PRE-INSERTION PREPARATION

Pre-Insertion Checklist

- Confirm clinical indication for UVC
- Calculate insertion depth (formula/weight table)
- Prepare sterile field, equipment & assistant
- Select ultrasound probe(s) in advance
- Optimise machine settings (depth 4–6 cm)
- Define roles: operator + POCUS assistant

Probe selection: Start with phased-array/curvilinear → switch to high-frequency linear (8–18 MHz) for IVC–RA tip confirmation

Linear 8–18 MHz	Phased-array 6–12 MHz	Curvilinear 5–12 MHz
IVC–RA tip confirmation. High resolution, shallow depth.	Subcostal cardiac views; detects intracardiac extension.	Broad FOV for intrahepatic trajectory & portal sinus anatomy.

PHASE 2 — UVC INSERTION & ANATOMICAL PATHWAY

Sterile Blind Insertion of UVC
Advance to calculated depth · Confirm free blood aspiration

Expected Anatomical Pathway: UV → IVC–RA Junction

Umbilical Vein (UV)	→	Portal Sinus	→	Ductus Venosus	→	IVC	→	IVC–RA Junction
		⚠ Most common deviation site		Confirm entry on US				✓ Target position

PHASE 3 — THREE-VIEW POCUS CONFIRMATION PROTOCOL

Structured Three-View POCUS Protocol (perform in sequence)

View 1 — Transverse Abdominal ("SITUS VIEW")	View 2 — Subcostal Longitudinal ("REFERENCE VIEW")	View 3 — Coronal / Oblique Liver ("HEPATIC VIEW")
Probe: below costal margin, marker → infant's left<br>**Confirm:** Aorta left of spine, IVC right of spine<br>⚠ Heterotaxy: situs may be reversed — check anatomy before advancing	**PRIMARY TIP LOCALISATION VIEW**<br>Probe: below xiphisternum, sagittal, marker → infant's head<br>**Confirm:** Bright echogenic tip at IVC–RA junction, not within RA itself	Probe: oblique subcostal/intercostal<br>**Confirm:** Ductus venosus entry into IVC; use when views 1–2 inconclusive.<br>Most reliable confirmation of ductus venosus passage

If tip not visualised: Probe Angulation	If tip not visualised: Saline Flush	Image Optimisation
Adjust probe angle to improve catheter echogenicity and tip visibility	A bolus of saline produces microbubbles — confirms intravascular position in real time	Depth 4–6 cm · Adjust gain (vessels black, tip bright) · Avoid probe compression of IVC

◆ Where is the UVC tip? ◆

PHASE 4A — TIP POSITION CLASSIFICATION & IMMEDIATE MANAGEMENT

☑ Optimal — IVC–RA Junction	⚠ Portal Sinus — Low Midline	△ Intracardiac — High / Within RA	⃠ Portal/Hepatic Branch — Malposition
**Appearance:** Echogenic tip at cavoatrial junction, just below lower RA border<br>**Risk:** Lowest complication risk<br>**Action:** ✔ Safe to use as intended. Rescan within 24 hours	**Appearance:** Tip below diaphragm, not entering IVC; midline on X-ray but portal sinus on US<br>**Risk:** Portal venous thrombosis; hepatic injury; unsafe for infusion<br>**Action:** ⟳ Reposition under POCUS (see Phase 4b)	**Appearance:** Tip in right atrium; moves with cardiac cycle<br>**Risk:** Arrhythmia; pericardial effusion; tamponade<br>**Action:** ⇩ Withdraw into IVC under continuous US guidance immediately	**Appearance:** Tip deviating laterally into portal/hepatic vein or parenchyma<br>**Risk:** Hepatic necrosis; thrombosis; extravasation<br>**Action:** ⃠ Do NOT use. Remove and re-site immediately

PHASE 4B — POCUS-GUIDED CORRECTION OF PORTAL DEVIATION

Portal Deviation Correction Protocol — when tip enters the portal system	
**Step-by-step approach:**<br>1. Use curvilinear/phased array to visualise portal sinus, ductus venosus & IVC together<br>2. Apply gentle external liver compression near portal sinus — transiently alters intrahepatic vein angle<br>3. Partially withdraw catheter, re-advance slowly under direct US view<br>4. Confirm passage through ductus venosus into IVC on all three views	**Important safeguards:**<br>• Do NOT use excessive force — resistance may indicate unfavourable anatomy or partial ductus venosus closure<br>• Avoid "railroading" a second catheter alongside existing UVC — increases infection & vascular injury risk<br>• If safe redirection cannot be achieved → remove and re-site

☑ UVC confirmed at IVC–RA junction on all three views → Safe to use

PHASE 5 — POST-INSERTION SURVEILLANCE PROTOCOL

Serial POCUS Surveillance — Migration occurs in 50–90% of neonates within the first 72 hours

When to Rescan	Indication / Rationale
✔ **Routine: Within 24 hours**	Even after apparently optimal initial placement — highest risk period for migration
✔ **Routine: Every 48 hours**	While UVC remains in situ — pragmatic safety surveillance
● **Triggered: After any manipulation**	After repositioning or documented change in external catheter length
● **Triggered: Clinical deterioration**	Unexplained hypotension, new arrhythmia, or change in catheter function
● **Triggered: Abdominal signs**	New abdominal distension or isolated ascites → assume catheter-related extravasation until excluded
● **Triggered: Loss of blood return**	Previously aspirated blood return no longer obtainable

PHASE 6 — GOVERNANCE, TRAINING & DOCUMENTATION

Training & Competency
Structured educational pathway; >10 supervised scans required to achieve procedural competency (Kochan et al.)

Documentation
Structured scan record for each assessment: tip position, view quality, action taken, operator, date/time

Image Storage & Audit
Secure image archiving; peer review of saved images; regular audit within local governance framework

Framework requirement (ESPNIC, AAP, Canadian Paediatric Society): POCUS must be delivered within institutional frameworks defining scope of practice, competency thresholds, and mechanisms for image review. Integrate into existing UVC pathways — not as a parallel workflow.

EVIDENCE SUMMARY

**93.3%**<br>POCUS sensitivity vs 66.7% radiography (Michel et al.)	**RR 0.51**<br>Reduced malposition risk with POCUS vs radiography (Mohamed et al. 2025 meta-analysis, n=1,322)	**50–90%**<br>Neonates with UVC migration within the first 72 hours (Dubbink-Verheij, Franta et al.)	**486**<br>Radiographs avoided with daily POCUS surveillance in one prospective cohort (Xie et al.)	**9.6%**<br>Malposition rate with real-time US guidance vs 75.9% without (D'Andrea et al.)

UV = Umbilical Vein · DV = Ductus Venosus · IVC = Inferior Vena Cava · RA = Right Atrium · POCUS = Point-of-Care Ultrasound

Supplementary Table 1

Comparison of radiography and POCUS for UVC tip localisation and surveillance.

This table summarises the principal differences between thoracoabdominal radiography and point-of-care ultrasound (POCUS) for umbilical venous catheter tip confirmation and ongoing surveillance, drawn from the comparative evidence reviewed in the main manuscript.

Supplementary Table 1. Comparison of radiography and point-of-care ultrasound (POCUS) for umbilical venous catheter tip localisation and surveillance in neonate

Parameter	Radiography	POCUS
**Method of tip localisation**	Indirect; inferred from vertebral and diaphragmatic landmarks.	Direct; tip visualised at the IVC–RA junction.
**IVC–RA localisation**	Limited; varies with projection and patient positioning.	Definitive; cavoatrial junction seen directly
**Portal tract malposition**	Unreliable; often misclassified as “low midline”.	Reliably identified; catheter traced along vessel through portal sinus and ductus venosus.
**Migration detection**	Requires repeat films.	Immediate bedside reassessment, repeatable as often as clinically required.
**Real-time guidance**	Not possible.	Available during insertion and manipulation.
**Radiation exposure**	Cumulative, particularly with serial imaging.	None.
**Diagnostic accuracy**	Moderate pooled sensitivity and specificity [7].	Higher accuracy at the IVC–RA junction[8]; halves malposition rate vs radiography in pooled meta-analysis[24].
**Operator dependence**	Standardised acquisition; interpretation relies on trained personnel.	Operator-dependent at both acquisition and interpretation; accuracy depends on probe handling, image optimisation, and artefact recognition (e.g. side-lobe ghosting at the cavoatrial junction) [25].
**Training requirement**	Established radiographic training pathway; widely standardised across centres.	Requires structured neonatal POCUS training, with approximately ten supervised scans typically needed to achieve competency [10].

Supplementary Appendices 1–3

The bedside POCUS UVC checklist (Appendix 1), documentation template (Appendix 2), competency framework (Appendix 3), and the practical guidance flowchart (Supplementary Figure 2) are presented on the following pages in A3 landscape format for clinical readability.

After this page, the document switches to A3 landscape orientation for the flowchart and appendices content.

SUPPLEMENTARY APPENDIX 1

Bedside POCUS UVC Checklist

BEFORE USE OF UVC

- ☐ Indication confirmed
- ☐ Insertion depth calculated
- ☐ Sterile insertion completed
- ☐ Blood aspirates freely

POCUS CONFIRMATION

- ☐ Situs / portal sinus view obtained
- ☐ Subcostal IVC–RA view obtained
- ☐ Tip visualised *(see it, move it & flush it)* with good aspiration and flushing of UVC.
- ☐ Tip confirmed at IVC–RA junction

DECISION

- ☐ Safe **to use**
- ☐ Needs **adjustment**
- ☐ Unsafe **— do not use**

RE-CHECK PLANNED WITHIN 24 H

☐ Yes ☐ Not applicable

SUPPLEMENTARY APPENDIX 2

UVC POCUS Documentation Template

SCAN DETAILS

- Date / time of scan:
- Operator:
- Probe(s) used:

VIEWS OBTAINED

- ☐ Situs
- ☐ Subcostal IVC
- ☐ PSAX

FINDINGS & ACTION

- Tip location:
- Action taken:
- Final status:

IMAGE RECORD

- Images saved: ☐ Yes ☐ No

SUPPLEMENTARY APPENDIX 3

Minimum Competency Framework for UVC POCUS

To be signed off for independent practice:

MANDATORY TRAINING

- ☐ Attend formal neonatal POCUS teaching
- ☐ ≥10 supervised UVC scans

DEMONSTRATED ABILITY TO

- ☐ Identify course
- ☐ Identify tip
- ☐ Recognise malposition
- ☐ Guide safe manipulation

SIGN-OFF

- ☐ Local governance sign-off

Trainee — name & signature

Supervisor — name, role & signature

Date
